# Feasibility of transapical aortic valve replacement through a left ventricular apical diverticulum

**DOI:** 10.1186/1749-8090-8-3

**Published:** 2013-01-07

**Authors:** Enrico Ferrari, Mathieu Van Steenberghe, Jegaruban Namasivayam, Denis Berdajs, Lars Niclauss, Ludwig Karl von Segesser

**Affiliations:** 1 Department of Cardiovascular surgery, CHUV, University Hospital of Lausanne, Rue du Bugnon 46, Lausanne, CH-1011, Switzerland

**Keywords:** Aortic valve replacement, Transcatheter aortic valve implantation, Left ventricular apical diverticulum

## Abstract

Transapical aortic valve replacement is an established technique performed in high-risk patients with symptomatic aortic valve stenosis and vascular disease contraindicating trans-vascular and trans-aortic procedures. The presence of a left ventricular apical diverticulum is a rare event and the treatment depends on dimensions and estimated risk of embolisation, rupture, or onset of ventricular arrhythmias. The diagnosis is based on standard cardiac imaging and symptoms are very rare. In this case report we illustrate our experience with a 81 years old female patient suffering from symptomatic aortic valve stenosis, respiratory disease, chronic renal failure and severe peripheral vascular disease (logistic euroscore: 42%), who successfully underwent a transapical 23 mm balloon-expandable stent-valve implantation through an apical diverticulum of the left ventricle. Intra-luminal thrombi were absent and during the same procedure were able to treat the valve disease and to successfully exclude the apical diverticulum without complications and through a mini thoracotomy. To the best of our knowledge, this is the first time that a transapical procedure is successfully performed through an apical diverticulum.

## Background

Transcatheter aortic valve replacement (TAVR) is an established minimally invasive technique for patients with severe symptomatic aortic valve stenosis and surgical high-risk profile. Predominant accesses are the transapical and the transfemoral ones, but, recently, also the trans-subclavian and the trans-aortic access have been employed to perform successful transcatheter aortic valve procedures. However, severe atherosclerosis, heavy calcifications, small diameters and tortuosities limit the trans-vascular and the trans-aortic access, whereas a left ventricular dysfunction, presence of apical thrombi and anatomical left ventricular anomalies (such as an aneurysm or an apical diverticulum) can constrain the transapical approach [1]. Recently, we already demonstrated that TAVR can be safely performed through a chronic left ventricular apical aneurysm, as long as apical thrombi are absent [2]. In this new report, and for the first time ever, we show the proof that a transapical aortic valve procedure can be safely performed through a left ventricular apical diverticulum without apical thrombi, and that the apical diverticulum can be excluded during the same procedure.

## Case presentation

**Figure 1:**
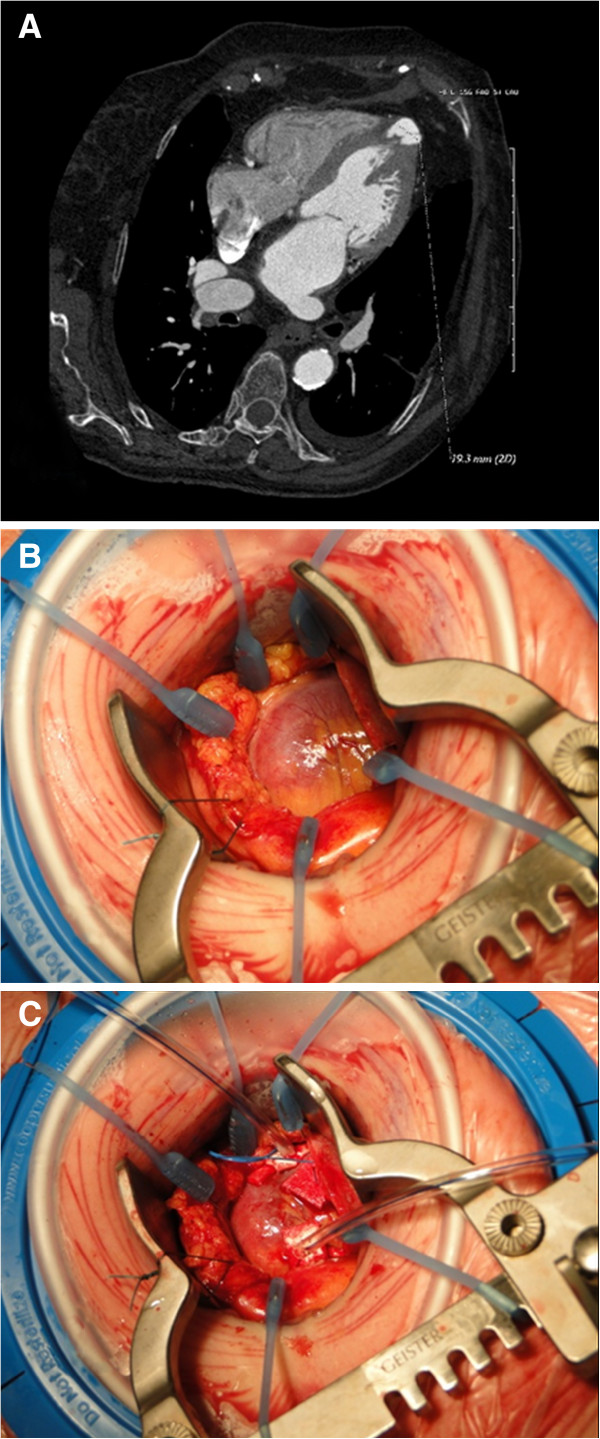
**A) A computed tomography scan showing the left ventricular apical diverticulum: note the absence of thrombi. B)** Intraoperative view of the cardiac apex: macroscopically, there is no evidence of a diverticulum, whereas the palpation reveals a soft portion. **C)** Careful preparation of a double pledgeted 3–0 Prolene purse-string suture around the diverticulum.

An 81 year old female with severe symptomatic aortic valve stenosis was screened for a transcatheter aortic valve procedure. She carried several comorbidities: severe obstructive respiratory disease, peripheral vascular disease with small calcified femoral arteries, small subclavian arteries and diseased ascending aorta, contraindicating all trans-vascular approaches. Moreover, a diffuse coronary sclerosis without significant stenosis was diagnosed, and the patient also suffered from a chronic kidney failure so that we opted for a transapical procedure fully guided by transesophageal echocardiography without intraoperative angiographies. During the preoperative imaging assessment, we performed a computed tomography scan with low contrast that revealed the presence of a congenital apical diverticulum of the left ventricle without thrombi. Diameters were 13 mm and 16 mm (Figure 1A). The echocardiogram showed a severely degenerated and much calcified aortic valve with trans-valvular peak gradient of 53 mmHg, surface area of 0.6 cm^2^, left ventricular ejection fraction of 65% and pulmonary hypertension. The apical diverticulum was not visualized. The patient accepted a transapical aortic procedure through the left ventricular diverticulum and the calculated operative risk was 42% (logistic EuroSCORE).

**Figure 2:**
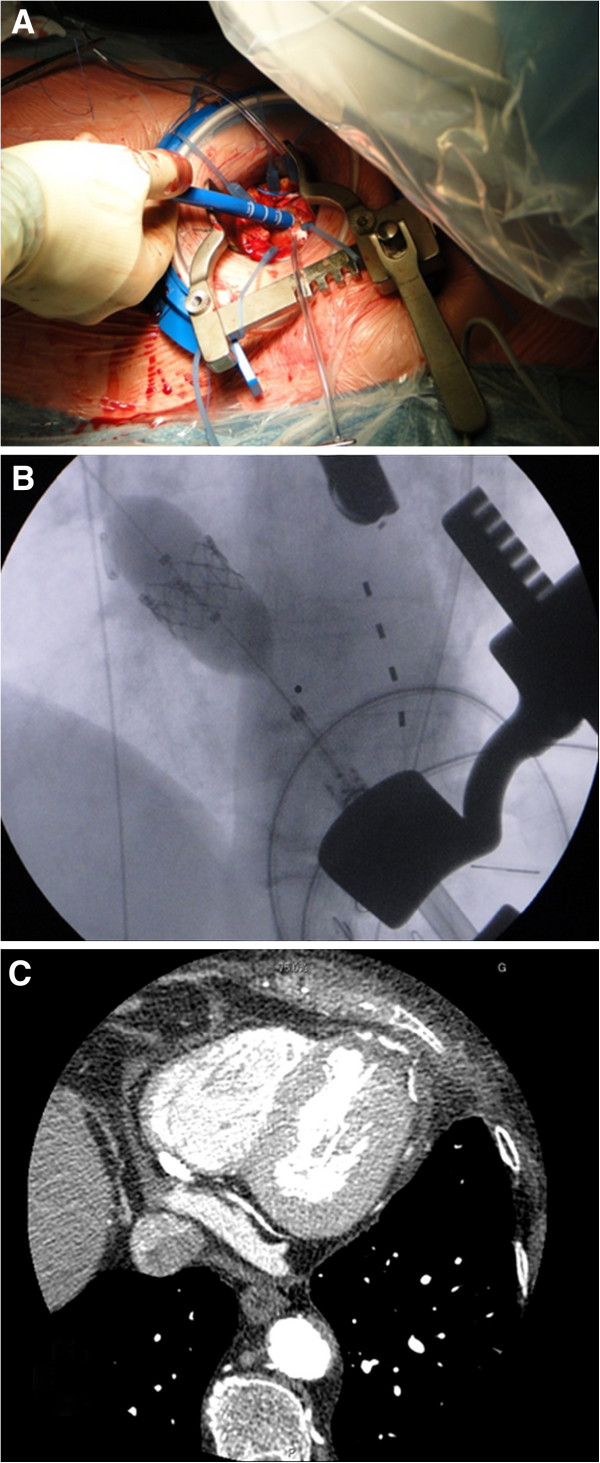
**A) Intraoperative view showing the introduction of the delivery system (Ascendra™ 2) in the apex. B)** Fluoroscopic view of the stent-valve deployment under rapid pacing. **C)** Postoperative computed tomography scan showing the good result with partial exclusion of the diverticulum.

The transapical stent-valve procedure was performed under general anesthesia and in the operating room. From a surgical point of view, the apical approach was uneventful: macroscopically (Figure 1B), we did not observed any external sign of the presence of the diverticulum, whereas the apical palpation revealed a thinner wall in a 2 cm^2^ wide region. There were no adhesions in the pericardium. A double pledgeted purse-string suture with 3–0 Prolene was carefully and successfully performed around that area (where the myocardium was ticker). Then, the delivery system (Ascendra™ 2) was introduced, uneventfully, in the left ventricle through the diverticulum (Figures 1C and 2A). Following the standard technique a 23 mm Sapien™ XT stent-valve (Edwards Lifesciences Inc., Irvine, CA) was successfully implanted with final trans-valvular mean and peak gradients of 10/4 mmHg (Figure 2B). Then, the delivery system was retrieved and the apical sutures were tided under rapid pacing. The entire procedure required 80 minutes to be performed and we did not experienced apical complications. A postoperative scan confirmed the stent-valve placement with absence of residual diverticulum in the apex (Figure 2C). The postoperative recovery was uneventful and the patient was discharged 8 days later.

## Conclusions

A left ventricular diverticulum is defined as an outpunching structure that contains endocardium, myocardium and pericardium and displays normal contraction. They are distinguished from the aneurysms which do not contract, have a fibrous wall and exhibit paradoxical motion. Earlier studies report a prevalence of diverticula in 0.4% or 3% of 750 cardiac necropsy cases [3,4]. They are congenital (in absence of history of injured myocardium), asymptomatic (except for rare cases of ventricular tachycardia), and most of them are placed in the apex [5]. There is no consensus about the treatment of this ventricular anomaly and the management should be tailored to the clinical characteristics of each patient, taking into consideration the onset of potential complications (embolization, rupture, ventricular arrhythmias) [6]. The surgical treatment consists of an excision and placement of a patch.

During a transapical transcatheter aortic valve replacement, the apex is prepared with two purse-string sutures and then punctured in order to introduce the delivery system. Thus, in the presence of a diverticulum without intraluminal thrombi, and in the absence of good alternative vascular and accesses, the transapical approach appears to be adequate in order to treat, simultaneously, both the apical diverticulum and the aortic valve stenosis.

In our experience, we prepared two larger pledgeted purse-string sutures in order to detect good thick myocardium surrounding the diverticulum: using this stratagem, part of the diverticulum was successfully excluded when the sutures were tied and we did not experienced apical bleeding.

With regards to the postoperative management, we did not change our protocols but we performed a computed tomography scan to visualize the resulting apical anatomy. In conclusion, TAVR procedures can be safety and efficacy performed through a left ventricular apical diverticulum, in the absence of intraluminal thrombi.

## Consent

The patient gave his informed consent for publication.

## Competing interests

EF is consultant for Edwards Lifesciences.

## Authors’ contribution

All Authors equally contributed to this paper. All authors read and approved the final manuscript.
